# Thematic Foreword from the Guest Editor to the Special Issue Entitled “The Role of Interactions in Complexes, Clusters and Crystal Structures—Theoretical Analyses and Experimental Evidences”

**DOI:** 10.3390/ijms24076680

**Published:** 2023-04-03

**Authors:** Oleg V. Mikhailov

**Affiliations:** Department of Analytical Chemistry, Certification and Quality Management, Kazan National Research Technological University, K. Marx Street 68, 420015 Kazan, Russia; olegmkhlv@gmail.com

The world around us consists of a huge number and a wide variety of substances—both individual chemical compounds and their compositions (mixtures). Two factors play a decisive role in the presence of such diversity, namely, the nature of the atoms (nuclides) that make up these substances and the specificity of the interactions between atoms and/or more complex structural fragments formed by these atoms (in particular, molecules). At the same time, the second factor frequently plays a more important role than the first; for example, white (yellow) phosphorus P_4_ and yellow arsenic As_4_ are formed by atoms of different chemical elements but have the same type of spatial structure, with a cubic lattice, and in terms of the nature of the interaction between atoms, these two compounds are much more similar to each other than white phosphorus P_4_ and black phosphorus P_n_ formed by atoms of the same element but with a different spatial structure (cubic for white and rhombic (graphite-like) for black). With this example in mind, the list of such compounds does not end there and can easily include other examples. For this reason, one of the interesting and important scientific areas of modern physicochemistry is the identification of the specific mechanisms of interactions in already known substances and the prediction of such for those substances that have not yet been obtained but can exist in principle according to the data of theoretical calculations (primarily, quantum-chemical calculations).

Most clearly, the specificity of the interactions between the structural fragments of a chemical substance (atoms, molecules, ions, etc.) manifests itself in the form of the existence of three aggregate states for any of these substances, namely, solid, liquid, and gaseous (Table 1). In this regard, it should be emphasized that there are exactly three states of aggregation, no more and no less. The point is that two polar variants of matter structures are purely logically conceivable; in the first, the structural fragments of matter are rigidly fixed in space and time, while in the second, they can freely move in any direction of space and time without any restrictions. The first of these variants self-evidently corresponds to a solid state of aggregation; in this state, the substance has a fixed shape and volume. The second variant corresponds to the gaseous state, in which the substance has neither one nor the other. However, an intermediate variant in which the structural fragments of matter are no longer rigidly fixed in space/time, but the possibilities of their movement are very limited, is also possible; this variant corresponds to the liquid state. In this state, matter does not have a fixed shape but a fixed volume. The difference between these states is quite clearly visible already at the macro-level of the organization of matter and is recorded by our organs of vision. Nevertheless, within each of these three states of aggregation, two “substates” can be distinguished, which already manifest themselves mainly at the micro-level and are usually indistinguishable in outward appearance. In the case of solid-state materials, these are crystalline and amorphous, while in the case of those in a liquid state, they are anisotropic (more often called the liquid crystalline state or liquid crystals) and isotropic; lastly, in the case of a gaseous state, substances are below the critical temperature and above the critical temperature (Table 1). At the same time, the “substates” of the solid and liquid states of aggregation, albeit slightly, are still not identical in terms of the indicators indicated in Table 1, while in the case of a gaseous state, they are identical. Without going into further detail here, which is already beyond the scope of this editorial, it should be noted that the indicators presented in this table refer to virtually ANY substance, regardless of its chemical composition. The transitions between states of aggregation, as a rule, occur at strictly defined temperature and pressure and are accompanied by a sharp (jump-like) change in the physicochemical characteristics of substances (the only exceptions are the processes of sublimation and evaporation, which occur at any temperature and pressure). These parameters are determined by the nature of the substance and the nature of the interactions between its structural fragments (atoms, molecules, ions, etc.). In a solid state of aggregation, these are interactions between atoms, molecules, or ions that constitute the so-called nodes of the crystal lattice; in liquid and gaseous states, interactions occur between the molecules of the corresponding substances. The specificity of these interactions, in turn, is determined by the interactions within these structural fragments; in this regard, three main types can be distinguished, namely, electrostatic (Coulomb), magnetic, and van der Waals interaction (which, however, is also based on electrostatic interactions). Accordingly, five main types of bonds exist between these structural fragments: ionic, covalent, metallic, hydrogen, and van der Waals bonds.

Ionic bonds arise between atoms of chemical elements, the electronegativity of which greatly differs from each other (by at least 1.7 on the Pauling scale). In the process of the formation of any such bond, a significant transfer of electron density occurs from atoms with a lower electronegativity to atoms with a higher electronegativity; as a result, the first set of atoms acquire a positive charge, while the second, a negative charge, thus inducing an electrostatic interaction between them. This is a very strong bond (its energy varies from 170 to 1500 kJ/mol) [1].

Covalent bonds are formed due to the formation of electron pairs with different spins [2]. Each of these electrons, due to its own rotation, forms a miniature circular current, which in turn leads to the formation of miniature magnets. The poles of these magnets are opposite to each other, and thus attractive forces arise between them. Additionally, as the data of quantum-mechanical calculations reveal, the forces of attraction between such electrons are more significant than the forces of electrostatic repulsion between them. Covalent bonds are formed either between atoms of the same chemical element or between atoms of those elements whose electronegativity difference is not too significant. In the second case, to some extent, a shift in the electron density is also evident from atoms with lower electronegativity to atoms with higher electronegativity; thus, both magnetic and electrostatic interactions contribute to the chemical bond formed. In this context, it is worth noting that there are two variants for a covalent bond according to the method of formation: in the first, electrons are supplied by all the atoms participating in the interactions to form bonds (the so-called exchange interactions), while in the second, only a part of these atoms supply electrons (i.e., those that undergo the so-called donor–acceptor interactions, in which the atoms supplying electrons are donors, and their chemical bond partners are electron acceptors due to the presence of vacant orbitals, where the received electrons are placed). Running a little ahead in the course of our story, it should be noted that the second variant of covalent bonds is typical for the most extensive group of chemicals, united under the general name “coordination compounds” or “compléxes” (with emphasis on the second syllable). The energy of the covalent bond as a whole is slightly less than the energy of the ionic bond and ranges from 120 to 1100 kJ/mol.

The third of the above types of chemical bond—the metallic bond—owes its origin to electrostatic interactions, similar to the ionic bond already mentioned above, but it is generated through a significantly different mechanism than that underlying the formation of an ionic bond [3]. First, such a bond is, in principle, formed between atoms of the same chemical element; secondly, it is realized only in the solid aggregate state of matter; finally, it is always multicenter and multielectron, and for its implementation, at least tens (if not hundreds) of atoms are needed (by contrast, an ionic bond can be realized between only two atoms, as is the case, for example, in chloride potassium (KCl) or barium oxide (BaO)). The formation of a metallic bond requires the availability of atoms having relatively low electronegativity, which enables them to donate electrons fairly easily; in addition, the number of electrons must be much smaller than the number of orbitals that can participate in the formation of chemical bonds. When these conditions occur, a relatively small number of electrons bind together and form a significantly larger number of nuclides. As a result, a specific structure emerges, in which the positively charged ions of the corresponding chemical elements appear at the nodes of the crystal lattice, and electrons move freely in the interstitial space. Thus, the pronounced delocalization of the electron density within the entire structure occurs. Such a structure is typical for those substances that have long been collectively known as “metals” (hence called a metallic bond). The energy of a metallic bond is comparable in order of magnitude to the energy of covalent and ionic bonds and even exceeds it since it ranges from 200 to 2000 kJ/mol. It is well established that metals constitute the absolute majority of all the chemical elements included in the D.I. Mendeleev Periodic System; all s-, d- and f-elements belong to them, as well as some of the p-elements: Al, Ga, In, Tl, included in the III (XIV) group; and Sn, Pb (IV (XV) group), and Bi (V (XVI) group). In this context, it should be noted that the atoms of metal elements are capable of forming not only metallic but also covalent bonds with each other.

In several cases, the interactions associated with the formation of hydrogen bonds play a very significant role. The term “hydrogen bond”, however, should not be taken literally, i.e., as a bond formed with the participation of hydrogen atoms, if only because this bond is not identical to a covalent or ionic bond (similar to an ionic bond, however, it is also the result of electrostatic interactions) [4]. Notably, within the framework of classical concepts, this bond is formed with the participation of not a hydrogen atom but its positively charged ion H^+^ (proton), which arises from a hydrogen atom as a result of contact with atoms of chemical elements with very high electronegativity values, namely F, O, N, Cl, and (only occasionally) S. Currently, within the framework of the theory of molecular orbitals (MO-LCAO), hydrogen bonding is considered a special case of covalent bonding with electron density delocalization along the atomic chain and the formation of three-center four-electron bonds (for example, in H_2_F_2_) or even four-center five-electron bonds (for example, in H_2_O, where the proton of one water molecule is bonded to two oxygen atoms of two neighboring molecules).

The features of the hydrogen bond, due to which it is distinguished as a separate species, include its low strength; its prevalence and importance, especially in organic compounds; and having some side effects associated with the very small size of the proton (its radius is only 0.841 fm, while the radius of the hydrogen atom is 53 pm) and the absence of electrons. The presence of such bonds, despite their relatively low energy (up to 40 kJ/mol in the case of neutral molecules), significantly affects the properties of numerous chemicals; for example, they increase the boiling point, viscosity, and surface tension of liquids, and they are responsible for many of the other unique properties of water. These ties are far less strong than any of the previous three; their energy varies from 10 to 160 kJ/mol.

The last of the types of bonds mentioned above, the van der Waals bond, is realized between molecules, and it is this type of bond that, in many cases, is “responsible” for intermolecular interactions. The formation of these bonds, however, is also associated with the manifestation of Coulomb forces; in terms of the objects between which these forces occur, they are dipoles, i.e., systems consisting of two charges, equal in magnitude and opposite in sign, and located at a distance from each other that is very small compared with the distance to the observation point [5]. Dipoles are divided into two categories—permanent and induced—and accordingly, three types of van der Waals interactions are known: orientational, which occurs between permanent dipoles; inductive, occurring between a permanent dipole and induced dipoles; and dispersing, occurring between two induced dipoles. Owing to the last of these three varieties, the possibility of the existence of all three states of aggregation of inert gases (the structural units of which are not molecules but individual atoms), as well as several supramolecular compounds, is ensured. The bonds formed in the framework of the van der Waals interaction have very low energy (5–20 kJ/mol).

Along with the interactions indicated above, one cannot fail to note one more that stands apart, and although it does not yet noticeably manifest in chemical compounds, it does undoubtedly, and probably will for the foreseeable future, be formed. We are talking about the so-called “relativistic effect” [6], meaning that with an increase in the charge of the nucleus of an atom, the speed of the movement of electrons surrounding its nucleus becomes commensurate with the speed of light (c). As a consequence, a type of compression force exists between the electronic layers of the atom. First of all, most electronic layers located close to the nucleus of the atom (that is, with the lowest energy) are subject to this compression; however, to one degree or another, it also affects the higher electronic layers. According to the data of several quantum-mechanical calculations, for the elements of the sixth period, this effect becomes so significant that it begins to affect the outer electronic layers, the electrons of which take part in the formation of chemical bonds. This is also evident in the chemical properties of substances that include these elements. For chemical elements with a nuclear charge (Z) greater than 110, the influence of this factor is believed to be so great that it is expected to radically change the ideas about their physical and chemical properties, already established based on the Periodic Law of D.I. Mendeleev. Thus, there is reason to believe that simple substances formed by elements No. 112—copernicium (Cn) and No. 114—flerovium (Fl) will not only have a very low reactivity but, in terms of their chemical properties, are also close to noble gases such as radon Rn (Z = 86). Moreover, some researchers, based on theoretical calculations, believe that copernicium in the form of a simple substance is already gaseous at room temperature. However, it should be noted that there is also a directly opposite opinion on this subject, which posits that Cn, like its group II analogue mercury (Hg), is a volatile liquid at room temperature, with a density close to that of mercury [7]. In essence, the influence of this factor on the chemical properties of substances formed by superheavy atoms is only just beginning to be studied, and even then only at a theoretical level, since experimental data on the physicochemistry of the chemical elements corresponding to them are currently very few.

The types of interactions listed above play a special role in compounds located in the “boundary zone” between organic and inorganic substances, namely, in mono- and polynuclear coordination compounds (complexes) with chelate and macrocyclic ligands (both organic and inorganic). These interactions are particularly significant in the so-called clusters containing the structural unit with at least one covalent bond between atoms of metal elements. An unambiguous and generally accepted definition of the terms “coordination compound” and “compléx” has not yet been developed [8,9]. Moreover, even among chemists, there is no consensus as to whether these two terms should be considered synonymous with each other.

In our opinion, the most general definition of the term “compléx” indicates that a coordination compound or compléx is a chemical compound in which the distinguishing feature is the presence of at least one so-called central atom (complexator), surrounded in a certain way by some chemical structural formations (so-called ligands) capable of independent existence and heterolytic cleavage from it. At least part of the bonds of ligands with the complexator is realized according to the donor–acceptor mechanism. The concept of “cluster” is also ambiguous. This term was first proposed by F.A. Cotton back in the early 1960s [10], and initially, it was understood only as chemical compounds in which the presence of at least one covalent metal–metal bond was postulated (the simplest examples include dimercurium(I) dichloride (calomel) (Hg_2_Cl_2_) and dicopper(II) tetraacetate (Cu_2_(CH_3_COO)_2_) with Hg–Hg and Cu–Cu bonds, respectively). It is such an interpretation of this term (that is, in its original meaning according to F.A. Cotton) that will be used in the future. It should be noted that the word “cluster” subsequently received a broad interpretation, in chemistry (where it began to be used to designate associates in which not only metal–metal covalent bonds but also even metal atoms, for example, “water clusters”, were absent), as well as in those branches of science that are practically not related to chemistry, for example, in astronomy, economics, and linguistics [11]. On the other hand, in order to eliminate the above ambiguity in the definition of the term “cluster”, we consider it useful to use the term “metal cluster”, as suggested in a recently published article [12]. In contrast to the previous two, the concept of the third key object of study, which is presented in the title of this Special Issue, namely “crystal”, is more clearly defined: A crystal or crystalline solid is a solid material in which its constituents (such as atoms, molecules, or ions) are arranged in a highly ordered microscopic structure, forming a crystal lattice that extends in all directions [13]. This definition is used in chemistry; however, in a specialized branch of science involving the intersection of physics, chemistry, and mathematics, known as crystallography, another definition is adopted, given by the International Union of Crystallography. Based on this definition, a material is a crystal if it has a predominantly sharp diffraction pattern [14]. Crystals can form a wide variety of substances, including those that are the key objects of this Special Issue, and the types of interactions at the micro- and nano-levels that determine their existence can be very different and lead to the formation of any of the abovementioned bonds: ionic (in NaCl crystals), covalent (in boron nitride (borazon) BN crystals), metallic (in Fe crystals), and van der Waals bonds (in Ar crystals). It is not uncommon for crystals to have not one but a greater number of types of interactions (and thus bonds) between their constituent structures. Therefore, in crystals of sodium silicate(IV) (Na_2_SiO_3_), ionic bonds are realized between the sites of its crystal lattice, consisting of Na^+^ and SiO_3_^2−^ ions, and within SiO_3_^2−^ ions, covalent bonds between oxygen and silicon atoms; in crystals of the coordination compound, dioxygenyl hexafluoroplatinate(V) O_2_[PtF_6_], ionic bonds are realized between the sites of its crystal lattice, consisting of O_2_^+^ and [PtF_6_]^−^ ions, within the framework of the O_2_^+^ ion. In parallel, covalent bonds are generated between oxygen atoms, within the framework of the [PtF_6_]^−^ ion, while quasi-ionic bonds emerge between platinum atoms and fluorine. In ice (H_2_O) crystals, van der Waals bonds are realized between its lattice nodes, and within the content of these nodes, covalent and hydrogen bonds manifest between hydrogen and oxygen atoms. The identification of such interactions in substances of these types is very important in branches of modern chemical science such as coordination, organometallic, and supramolecular chemistry, since it allows researchers to study different concentrations of these substances and thereby purposefully control both their synthesis and their physicochemical properties. It seems to us that, currently, at least three problems can be established, which are one way or another related to the specifics of interactions in complexes, clusters, and crystals:

- *The first problem is associated with the development of new methods and improvement in the existing experimental physicochemical methods for studying the structure of a substance.* Such methods involve the analytical signals allowing researchers to determine the very nature of the presence (or absence) of a specific type of interaction between the corresponding structural fragments of a substance (atoms, molecules, crystal lattice nodes, etc.), as well as the quantitative parameters characterizing this interaction. This problem, however, is more related to improvement in the appropriate instrumentation for the implementation of methods such as UV–VIS, EXAFS, XRD, ESR, NMR spectroscopy, etc., which have long been the main methods for studying the molecular, electronic, and crystal structures of matter. Such methods should involve specialists in the field of instrumentation; nevertheless, the participation of those researchers who use these methods in their research will undoubtedly be useful.
- *Another problem involves the interpretation of experimental data using the theoretical concepts underlying the above physicochemical methods.* To date, a significant array of methods for processing the experimental data obtained using various physicochemical techniques have already been established; however, there are still several unresolved issues. In particular, it is still a significant challenge to obtain reliable information about the spatial structure of molecules and/or crystals of those substances for which, for some reason, it is not possible to obtain single-crystal samples using EXAFS and XRD data. This problem is especially relevant for the three “C” mentioned in the title of this Special Issue (complexes, clusters, and crystals);
- *The third problem is related to improving theoretical (and, above all, quantum-chemical) methods for describing the specifics of each of the above interactions.* Currently, a significant number of quantum-chemical methods exist for calculating various chemical compounds, both semi-empirical and non-empirical (ab initio), which allow researchers to calculate the quantitative parameters associated with the corresponding type of interactions, with varying degrees of reliability. Two main groups of these methods are commonly known: The techniques of the first group involve the determination of the wave functions of the corresponding molecular orbitals, while those of the second group involve the determination of the electron density arising in these orbitals. The second variant, generally known under the umbrella of density functional theory (DFT) [15], has now become more widespread, which makes it possible to obtain sufficiently reliable data on the molecular and electronic structures of substances that are generally in good agreement with experiments and have relatively low time and energy costs associated with conducting calculation. Currently, at least dozens of versions of DFT differing in their functionals and basis sets have already been proposed by various authors. Notably, however, one functional describes the parameters of the molecular structure of complexes and metal clusters relatively, while the other describes the mutual arrangement and energies of their energy levels, and the third describes the thermodynamic parameters. This variety implies that there is no universal version of DFT that would be suitable “for all occasions”. The same, in essence, also applies to the quantum-chemical methods of the first group.

Although there are currently numerous studies indicating very significant progress in solving each of these three problems, many questions still remain unanswered; thus, it would be premature to claim that its potential has already been fully revealed. The papers amassed in this Special Issue of the *International Journal of Molecular Sciences* contribute to the further development of this scientific context, which has been, and always will be, a very important and significant area of modern chemical science.

## Figures and Tables

**Table 1:** Aggregate states of matter and key qualitative characteristics of their structures. The sign (+) means the presence of the corresponding property, and the sign (−) means its absence.

State of Aggregation	Availability of Rigid Structure	Availability of Short-Range Order	Availability of Long-Range Order	Availability of Anisotropy of Properties
Solid (crystalline)	**+**	**+**	**+**	**+**
Solid (amorphous)	**+**	**+**	**−**	**+**
Liquid (anisotropic)	**−**	**+**	**−**	**+**
Liquid (isotropic)	**−**	**+**	**−**	**−**
Gaseous (below the critical temperature)	**−**	**−**	**−**	**−**
Gaseous (above the critical temperature)	**−**	**−**	**−**	**−**
